# Evaluation of the antibacterial potential in shoot and root extracts of *Eryngium campestre* with emphasis on Egyptian ecotype

**DOI:** 10.1038/s41598-025-05193-9

**Published:** 2025-07-16

**Authors:** Salma Hassan Zaki, Dina Ahmed Selim, Noha Khalifa Abo Aasy, Shacker Helmi

**Affiliations:** 1 Department of Environmental Studies, Institute of Graduate Studies and Research, Alexandria University, Alexandria, Egypt; 2 Department of Pharmacognosy, Faculty of Pharmacy, Alexandria University, Alexandria, Egypt; 3 Department of Industrial Pharmacy, Faculty of Pharmacy, Alexandria University, Alexandria, Egypt

**Keywords:** *Eryngium campestre* ecotypes, Antibacterial, Multi drug resistant, Fractionation, Ethanolic crude extract, Microbiology, Plant sciences

## Abstract

The antibacterial potential of Egyptian *Eryngium campestre* (*E. campestre*) was evaluated by investigating the growth inhibition of ethanolic shoot and root crude extracts and their solvent fractions against nine bacterial species, revealing a selective differential effect. Phytochemical screening of crude extracts identified the presence of flavonoids, alkaloids, tannins, saponins, phenols, terpenoids, steroids, and coumarins, suggesting their potential contribution to the observed antibacterial effects. Notably, the ethyl acetate fraction of the shoot and the petroleum ether fraction of the root demonstrated the highest activity, inhibiting the growth of all tested bacteria. Comparative analysis with other ecotypes highlighted variations in their antibacterial potency. These findings position the Egyptian *E. campestre* ecotype as a promising source for pharmaceutical industry, particularly for developing biocontrol agents against bacterial infectious diseases, including multidrug-resistant strains.

## Introduction

Nowadays, a lot of current antibiotics are ineffective due to the extensive, inappropriate, irregular, and indiscriminate uses, accordingly many of bacterial species developed resistance to synthetic antibiotics^1–3^. Therefore, new classes of effective antibacterial substances are needed. Wild flora containing active phytochemical compounds with antibacterial potential has been widely explored^4,5^. The genus *Eryngium* of the family Apiaceae includes about 250 wild plant species with cosmopolitan distribution, mainly in temperate zones of all continents^6,7^. The pharmacological effects of *Eryngium* species were found to be due to the presence of flavonoids, phenolic acids, high triterpenoid saponin content, coumarin derivatives, acetylenes, rosmarinic acid chlorogenic acid, and sterols in some *Eryngium* species^8–11^. The Egyptian ecotype of *E. campestre* species inhabiting the western desert of Egypt was noticed to be resistant to all common plant pathogens including bacteria, fungi, and insects. This field observation led to investigating plant phytochemistry starting from testing the potential of its crude extract as well as its solvent fractions in inhibiting growth of several bacterial species. It was reported that the polyphenolic compounds in the whole plant extract of *E. campestre* have an antibacterial efficacy in controlling several plant pathogens^12^. It has been reported that the Egyptian ecotype of *E. campestre* extracts were found to be anti-Alzheimer, and antioxidant^13^.

Two objectives of this study are: testing the activity of the shoot and root ethanolic crude extracts of *E. campestre* and five solvent fractions of each as bacterial growth inhibitors as well as the minimum inhibitory concentration (MIC) of the crude extracts; and identifying active chemical compounds present in the ethanolic crude extracts of the shoot and root systems.

## Materials and methods

### Plant identification and floristic characterization

Samples were collected by Prof. Shacker Helmi, professor of Environmental Studies at Environmental Studies department of the Institute of Graduate Studies and Research, Alexandria University. He also identified the plant samples using Täckholm^14^ key flora of Egypt to be *E. campestre*. The plant collection adhered to all ethical guidelines, and permission was obtained from the Institute of Graduate Studies and Research at the University of Alexandria, Egypt. Herbarium sheet is available at the herbarium of the Botany department of the Faculty of Science Cairo University. It is a perennial, herbaceous flowering plant, and stout with rigid branches. Leaves are thick, coriaceous, blue green or sometimes yellow green^15^. The wild Egyptian ecotype grows in the western desert of Egypt, and its flowering season is spring. A voucher specimen (03–42-ALX) has been deposited at the Herbarium of the Department of Agricultural Botany, Faculty of Agriculture Saba Basha, Alexandria University, Alexandria, Egypt.

### Plant material collection and preparation

Enough amount of the whole plant was collected from Borg El-Arab district, 60 Km west of Alexandria city during August 2022. Plant samples were kept in plastic bags and labelled. In the laboratory, shoot and root systems were carefully separated, cut off, washed, rinsed, air dried, and ground to fine powder using an electric grinder. The final dry plant material weighed 500 g of shoots and 250 g of roots was stored in plastic bags, labelled, and kept away from elevated temperature, humidity, and light for future work.

### Plant extraction

Plant crude extraction was conducted according to the maceration extraction method^16,17^. A hundred and fifty grams (150 g) of the dried powdered material of shoot were soaked in one liter (1 L) of 70% ethanol in a conical flask (2 L). The flask was shaken for one week in a shaking incubator (New Brunswick G25, Scientific CO., INC. U.S.A) at 100 rpm at room temperature of 25 ± 5 Ċ, the crude extract was filtered using Whatman no 1 filter paper. Ethanol solvent was refreshed using the same procedure for another couple of weeks and the weekly filtrates were successively added up to a final volume. The filtrates were evaporated under reduced pressure using rotary evaporator at 40 ºC and the same method was applied for root extraction. Shoot and root extracts yielded about 14 and 43 g respectively.

### Fractionation

**Fig. 1:**
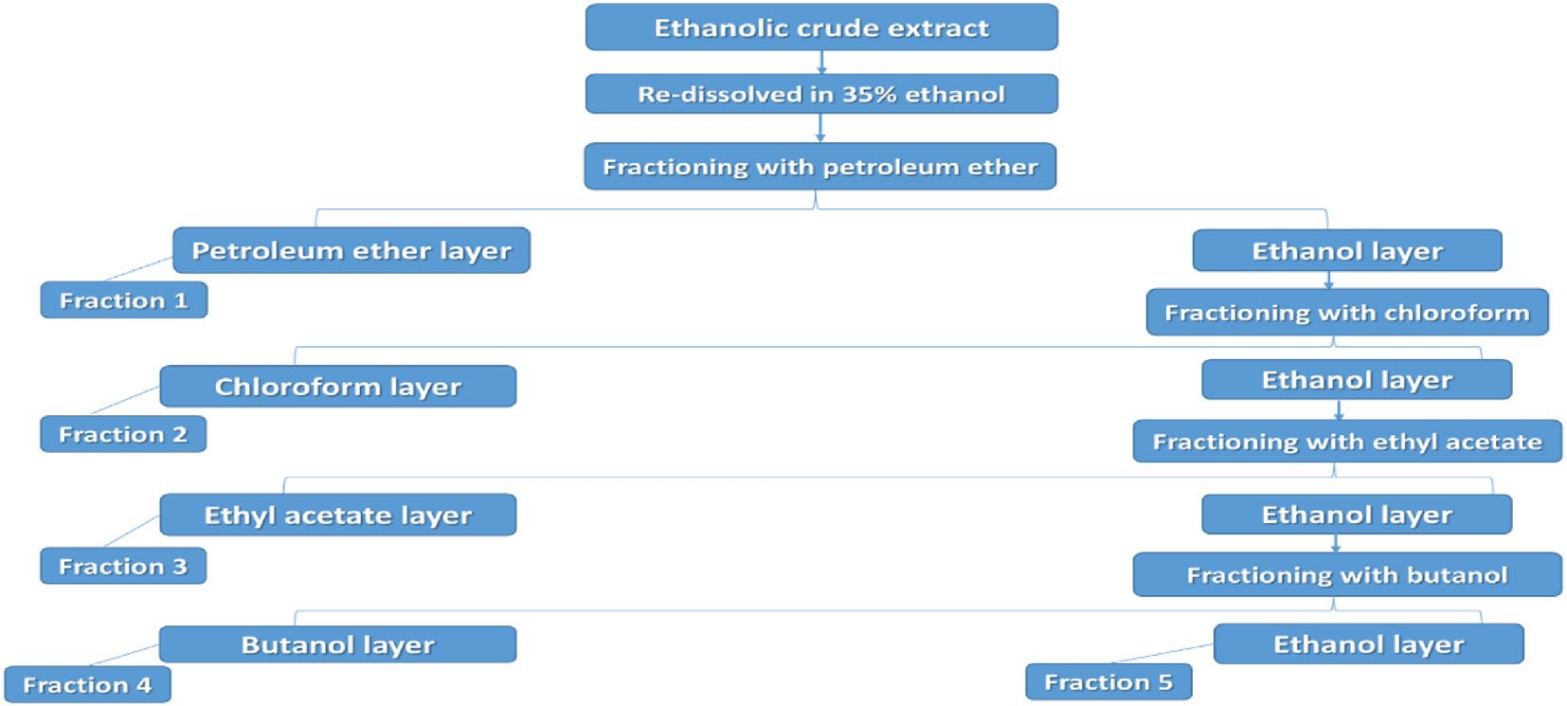
Flowchart of ethanolic crud extract fractionation procedure.

The ethanolic crude extract was fractionated using the successive solvent-solvent extraction procedure^18^. Briefly, semisolid shoot crude extract weighing 10 g was dissolved in 100 mL of 35% ethanol and poured into a 500 mL separating funnel, then 100 mL of petroleum ether were added to it. The mixture was vigorously shaken and allowed to stand until two distinct separate layers were observed. The two layers were separately collected in two conical flasks. One flask contained the petroleum ether fraction and the second contained the ethanol layer; the residual petroleum ether fraction was recovered by repeating the same step until the extracted petroleum ether fraction layer was colorless indicating the extraction of total petroleum ether fraction. In every round petroleum ether layer was collected successively in the same conical flask. After the collection of petroleum ether fraction, further fractionation was successively continued with the following solvents in the order of increasing polarity: chloroform, ethyl acetate, and butanol; the remaining volume contained ethanolic fraction. Each fraction was dried at 40 ºC under reduced pressure using rotary evaporator and fractions yield were 0.4752, 1.7423, 0.2896, 2.5240, and 4.9621 g for petroleum ether, chloroform, ethyl acetate, butanol, and ethanol 35%, respectively. The same procedure was followed for root crude extract weighing 20 g which yielded 0.531, 0.9481, 0.1088, 1.6159, and 15.9208 g for petroleum ether, chloroform, ethyl acetate, butanol, and ethanol 35%, respectively. The fractionation procedure is summarized in Fig. 1.

### Phytochemical screening

Phytochemical screening was carried out on both shoot and root ethanolic crude extracts of *E. campestre* using the methods described by Trease and Evans^19^ and Harbourne^20^ to detect the presence of secondary metabolites, such as flavonoids, alkaloids, tannins, saponins, phenols, terpenoids, steroids, and coumarin. These metabolites have been known for their potency in terms of antibacterial activity.

### Testing the solubility of the crude semisolid extract and its fractions in water

The semisolid shoot and root crude extracts and their fractions were found to be insoluble in water except the 35% ethanol fraction. Therefore, a mixture of 50% dimethyl sulfoxide (DMSO) and 2% tween 80 was prepared and used giving complete solubility without causing any inhibitory effect on bacterial growth as control.

### Tested bacterial species

Five different pathogenic reference bacterial species: three Gram negative namely *Klebsiella pneumoniae* ATCC 70,060, *Escherichia coli* ATCC 25,922, and *Pseudomonas aeruginosa* ATCC 27,853, and two Gram positive namely *Staphylococcus aureus* ATCC 29,213, and *Staphylococcus epidermidis* ATCC 12,228 were provided by the Microbiology unit, Faculty of Medicine, Alexandria University. In addition to two multi-drug resistant (MDR) species: *Klebsiella pneumoniae* and *Staphylococcus aureus* were provided by the Pathology laboratory, Alexandria general hospital and two Gram negative *Enterobacte*r *cloacae*, and *Acinetobacter baumannii*, were already available in the laboratory from previous work. These bacteria were maintained on glycerol and stored at −80 °C until to be activated for work.

### Bacterial activation and culture suspension

Bacterial activation and preparation of culture suspension were according to the Clinical and Laboratory Standards Institute CLSI guidelines^21^.

### Testing bacterial growth Inhibition

#### Agar well diffusion method

The Agar well diffusion method was used to test the bacterial growth inhibitory effect of the ethanolic crude extract and its fractions at a concentration of 100 mg/mL. Nine Muller Hinton Agar (M.H.A) plates were prepared; one for each bacterial species. Bacterial suspension of each species at density 1 × 10^8^ CFU were spread over the agar plate. Four wells were cut, one control in the center and three replicates, each well was 8 mm diameter. The wells were inoculated with 100 µL of crude extract and one inoculated with solvents mixture as control. This was repeated for all fractions. Plates were incubated at 37 °C overnight. The next day plates were examined for clear inhibition zones around wells and inhibition zone diameters were measured in mm.

#### Determination of minimum inhibitory concentration

According to the Clinical and Laboratory Standards Institute guidelines broth microdilution method was used to determine the minimum inhibitory concentration (MIC)^21^. Five species which gave positive growth inhibition in the agar diffusion method experiment namely: *Staphylococcus aureus* ATCC 29,213, *Staphylococcus epidermidis* ATCC 12,228, *Klebsiella pneumoniae* ATCC 700,603 *Staphylococcus aureus* MDR, and *Acinetobacter baumannii* were tested for their MIC. Two-fold serial dilutions were tested in a 96-well microtiter plate containing Muller Hinton broth medium (MHB) to obtain extract concentrations ranging from 50 to 0.18 mg/mL. Overnight cultures of the tested bacterial species in MHB were standardized to 0.5 McFarland turbidity standard. Uninoculated MHB was used as a sterility control, while inoculated MHB as a growth control and inoculated MHB with DMSO as a vehicle control. Plates were incubated at 37 °C overnight then MIC was determined by the change in colour using resazurin dye (0.015%)^22^. Experiments were performed in triplicates.

### Statistical analysis

Data were statistically treated using student t-test and the three-way ANOVA via Excel and Origin Lab software.

## Results

### Bacterial growth Inhibition of ethanolic shoot and root crude extracts

**Photo 1:**
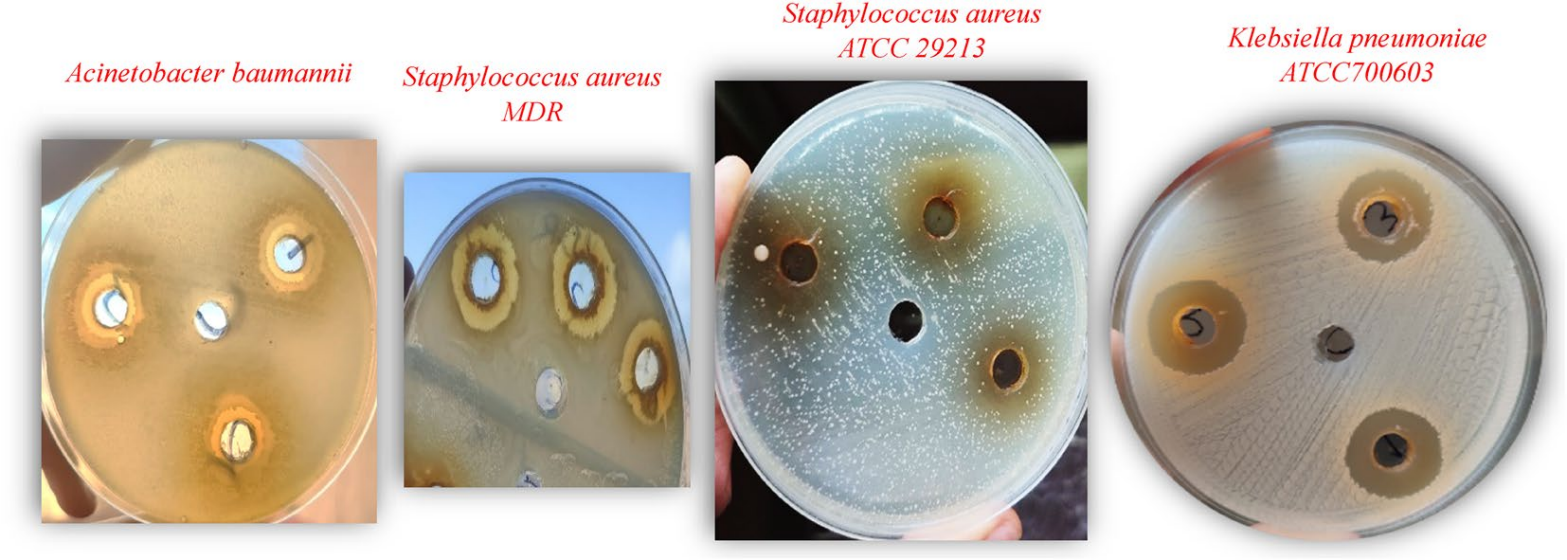
Growth inhibition zones of four bacterial species.

Out of the nine tested bacterial species treated with shoot and root ethanolic crude extracts each at a concentration of 100 mg/mL, only the growth of five species was inhibited but differed in two species between shoot and root extracts (Table 1). All the inhibited species showed variable sensitivity to the extracts (Photo 1). Paired t-test was carried out to test the difference in bacterial growth inhibition between the shoot and root ethanolic crude extracts as a factor affecting antibacterial activity which revealed insignificant difference.

**Table 1:** Selective and differential bacterial growth inhibitory effect of shoot and root crude extracts at 100 mg/mL.

**Tested bacterial species**	**Growth inhibition zone diameter (mm)**	
	**Plant part extract**	
	**Shoot**	**Root**
***S. aureus*** **ATCC 29213**	20±0.6	16± 1.7
***S. epidermidis*** **ATCC 12228**	8.0±0.0	12± 2.8
***K. pneumoniae*** **ATCC700603**	18±1.0	20± 0.0
***E. coli*** **ATCC 25922**	8.0±0.0	8.0±0.0
***P. aeruginosa*** **ATCC 27853**	8.0±0.0	8.0±0.0
***S. aureus*** **MDR**	19±1.0	8.0±0.0
***K. pneumoniae*** **MDR**	8.0±0.0	8.0±0.0
***A. baumanni***	15±0.0	18± 1.5
***E. cloacae***	8.0±0.0	8.0±0.0

Selective and differential bacterial growth inhibitory effect of shoot and root crude extracts at 100 mg/mL.

Data is expressed as mean (n= 3) ± SD (standard deviation).

Paired student t-test: t_cal_ = 0.5 < t_tab_ = 2.14, p = 0.05.

**Table 2:** Bacterial species minimum inhibitory concentration mg/mL values of shoot and root ethanolic crudeextracts of *E. campestre.*

**Tested bacterial species**	**Minimum inhibitory concentration (mg/mL)**	
	**Plant part extract**	
	**Shoot**	**Root**
***S. aureus*** **ATCC 29213**	6.25±0.0	3.75±0.0
***S. epidermidis*** **ATCC 12228**	N. I	6.25±0.0
***K. pneumoniae*** ** ATCC700603**	12.5±0.0	25±0.0
***S. aureus*** **MDR**	50±0.0	N. I
***A. baumanni***	12.5±0.0	50±0.0

Data is expressed as mean ± SD (n= 3).

N. I: No Inhibition.

### Minimum inhibitory concentration of crude extracts

Table 2 presents the minimum inhibitory concentrations (MICs) of shoot and root ethanolic crude extracts against five bacterial species that exhibited differential growth inhibition. The results clearly indicated differential inhibitory effect of shoot and root crude extracts as well as varying degrees of susceptibility of the bacterial species to the inhibitor.

**Table 3:** Means of bacterial growth inhibition zone diameters of solvent fractions of shoot and root ethanolic crude extracts at 100 mg/mL.

**Tested bacterial species**	**Plant part**	** Growth inhibition zone diameter (mm)**				
		**Solvent fraction**				
		**Petroleum ether**	**Chloroform**	**Ethyl acetate**	**Butanol**	**Ethanol**
***S. aureus ATCC 29213***	Shoot	18±0.0	17±0.0	13±1.0	22±3.0	8 ± 0.0
	Root	21±1.0	15±0.0	14±1.0	14±1.0	8 ± 0.0
***S. epidermidis ATCC 12228***	Shoot	18±0.0	20±0.0	13±0.0	20±0.0	8 ± 0.0
	Root	20±0.0	17±3	11±1.0	16±1.0	8 ± 0.0
***K. pneumoniae ATCC700603***	Shoot	20±0.0	8±0.0	12±0.0	18±1.0	8 ± 0.0
	Root	20±0.0	20±0.0	11±1.0	14±1.0	8 ± 0.0
***E. coli ATCC 25922***	Shoot	8±0.0	15±0.0	16±2.0	8±0.0	8 ±0.0
	Root	13±0.0	14±1.0	15±0.0	12±2.0	8 ± 0.0
***P. aeruginosa ATCC 27853***	Shoot	18±0.0	15±0.0	15±2.0	15±0.0	8 ± 0.0
	Root	15±0.0	15±0.0	8±0.0	15±2.0	8 ± 0.0
***S. aureus MDR***	Shoot	18±1.0	8±0.0	12±0.0	18±1.0	8 ± 0.0
	Root	17±0.0	8±0.0	11±1.0	14±2.0	8 ± 0.0
***K. pneumoniae MDR***	Shoot	8±0.0	8±0.0	11±1	8±0.0	8 ± 0.0
	Root	14±2.0	14±0.0	8±0.0	11±1.0	8 ± 0.0
***A. baumannii***	Shoot	8±0.0	8±0.0	15±1.0	19±1.0	8 ±0.0
	Root	20±0.0	20±0.0	16±1	24±1.0	14+1.7
***E. cloacae***	Shoot	8±0.0	8±0.0	13±1	11±1.0	8 ± 0.0
	Root	14±1.0	13±1.0	8±0.0	8±0.0	8 ± 0.0

Well diameter is 8 mm (means that there is no inhibition).

Data are means of three replicates (n = 3) ± standard deviation (SD)

### Bacterial growth inhibitory effect of solvent fractions

**Fig. 2:**
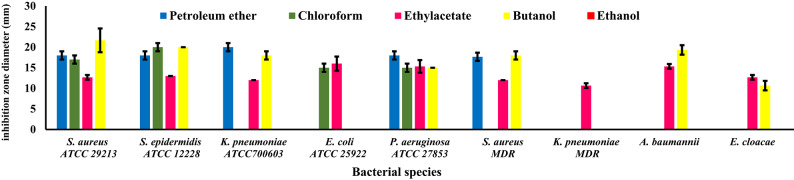
Means of bacterial growth inhibition zone diameters (mm) of shoot fractions.

**Fig. 3:**
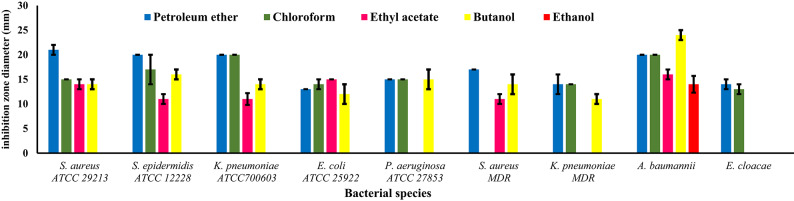
Means of bacterial growth inhibition zone diameters (mm) of root fractions.

**Fig. 4:**
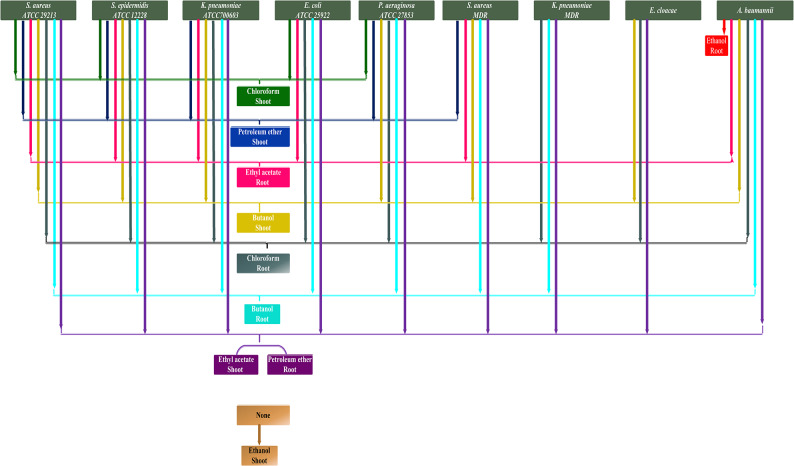
Bacterial growth inhibition selectivity and differential effect of extracted solvent fractions.

Table 3 and Figures 2 and 3 give the results of bacterial growth inhibition zone diameters of five shoot and root extracted fractions, against the same nine bacterial species. Tested bacteria differed in their degree of sensitivity to the growth inhibitory effect of solvent fractions. Regarding fractions (Fig. 4), petroleum ether of the root and ethyl acetate of the shoot were the most potent ones, inhibiting the growth of all nine tested bacterial species. On the contrary, shoot ethanol fraction was inefficient as it did not show any antibacterial activity. Root chloroform and butanol fractions inhibited the growth of all tested species except one: *S. aureus* MDR for chloroform and *E. cloacae* for butanol. On the other hand, shoot chloroform fraction inhibited four species except: *K. pneumoniae* ATCC700603, *S. aureus* MDR, *K. pneumoniae* MDR, *A. baumannii*, and *E. cloacae.* Petroleum ether fraction of the shoot inhibited the growth of five bacterial species: *S. aureus* ATCC 29,213, *S. epidermidis* ATCC 12,228, *K. pneumoniae* ATCC700603, *P. aeruginosa* ATCC 27,853, and *S. aureus* MDR and root ethyl acetate fraction inhibited the growth of all tested species except three: *P. aeruginosa* ATCC 27,853, *K. pneumoniae* MDR, and *E. cloacae.* Butanol fraction of shoot inhibited the growth of all tested species except two: *E. coli* ATCC 25,922 and *K. pneumoniae* ATCC700603. Root ethanol fraction showed the poorest activity as it inhibited only the growth of one species: *A. baumannii*. By comparing the antibacterial potency of plant parts, results illustrated that the root extract solvent fractions were more potent as all fractions inhibited more than one bacterial species. Statistical analysis (Table 4) proved that three factors: bacterial species, solvent fractions, and plant part, and their interactions are highly significant in their effect on bacterial growth inhibition. Furthermore, fractions generally outperformed crude extracts at the same concentration.

**Table 4:** Three-way analysis of variance: bacterial species, solvent fraction and part used and their interactions.

**Sources of variation**	**Df**	**F value**
Bacterial species	8	206.50436**
Solvent fraction	4	652.36337**
Part used	1	43**
Bacterial species * Solvent fraction	32	66.90225**
Bacterial species * Part used	8	78.54215**
Solvent fraction * Part used	4	112.57849**
Bacterial species * Solvent fraction * Part used	32	25.69513**
Model	89	93.77816**
Error	180	
Corrected Total	269	

*Significant (f-value at p ≤ 0.05).

**Highly significant (f-value at p ≤ 0.01).

**Table 5:** Bacterial growth inhibition selectivity of *E. campestre* worldwide ecotypes.

Ecotype	Plant part	Extraction method	Solvent used	extract	Extract tested concentration mg/mL	Tested bacteria	Inhibition zone diameter (mm ± SD)	Minimum inhibitory concentration (mg/mL)		Reference
Poland	Leaves and roots	Reflux	70% ethanol	crude	Not applicable	S. aureus ATCC 25,923	Not applicable	Leaves	Root	^9^
								1.9	0.9	
						B. subtilis ATCC 6633		1.5	1.9	
Romania	Aerial	Maceration	70% ethanol	crude	10	E. coli ATCC 25,922,	NI	NI		^23^
						P. vulgaris,	NI	NI		
						K. pneumoniae,	NI	NI		
						S. enterititidis,	NI	NI		
						P. aeruginosa ATCC 27,853,	25	0.007		
						S. aureus ATCC 29,213	NI	NI		
						S. epidermidis	NI	NI		

Ecotype	Plant part	Extractio method	Solvent used	extract	Extract tested concentration mg/mL	Tested bacteria	Inhibition zone diameter (mm ± SD)			Minimum inhibitory concentration (mg/mL)						Reference
Palestine	Whole plant	Serial exhaustive extraction	n-hexane 50% ethanol	crude	100		1 st aqueous	2nd aqueous	Organic	Aqueous						
						S. aureus 6538P	12	NI	10	10						^24^
						S. epidermidis 12,228	8	NI	8	NI						
						B. subtilis 6633	18	14	10	1						
						E. coli 8739	NI	NI	12	NI						
						P. aeruginosa 9027	NI	NI	NI	NI						
Serbia	Aerial parts and roots	ultrasonic bath for 30 min for organic solvents and hydro distillation for essential oils	water (H2O), methanol (MeOH), acetone (Acet) and ethyl acetate (EtOAc)	crude for aerial parts and essential oils (EO) for aerial and roots	Not applicable		Not applicable			H2O	MeOH	EtOAc	Acet	EO aerial	EO root	^25^
						S. aureus ATCC 6538				> 20	5	0.03	0.01	0.004	0.007	
						S. epidermidis ATCC 12,228				20	2.5	5	2.5	0.01	0.07	
						S. pyogenes ATCC 19,615				20	20	> 5	> 2.5	> 1.25	> 1.25	
						E. Faecalis ATCC 19,433				2.5	5	> 5	> 2.5	0.007	0.15	
						E. coli ATCC 8739				0.07	0.07	0.01	0.01	0.31	0.31	
						P.s aeruginosa ATCC 9027				20	20	> 5	> 2.5	1.25	1.25	
						P. mirabilis ATCC 12,453				10	5	0.007	0.004	< 0.0005	< 0.0005	
						K. pneumoniae ATCC 10,031				> 20	10	2.5	1.25	< 0.0005	< 0.0005	

Ecotype	Plant part	Extraction method	Solvent used	extract	Extract tested concentration mg/mL	Tested bacteria	Inhibition zone diameter (mm ± SD)	Minimum inhibitory concentration (mg/mL)	Reference
Algeria	aerial parts (including stems, leaves, and flower heads)	hydrodistillation	––	essential oil	Not applicable	E. faecalis ATCC 29,212,	35	0.125	^26^
						S. aureus ATCC 25,923,	35	0.125	
						S. aureus ATCC 33,862	21	0.25	
						S. aureus ATCC 29,213	20	0.25	
						B. subtilis ATCC 6633	N.I	NI	
						B. cereus ATCC 11,778	23	0.25	
						L. monocytogenes ATCC 19,115	N.I	NI	
						E. coli ATCC 25,922,	N.I	NI	
						K. pneumoniae ATCC 70,603,	N.I	NI	
						S. enteritidis ATCC 2453,	N.I	NI	
						P.s fluorescens ATCC 13,525	N.I	NI	

Ecotype	Plant part	Extraction method	Solvent used	extract	Extract tested concentration mg/mL	Tested bacteria	Inhibition zone diameter (mm ± SD)	Minimum inhibitory concentration (mg/mL)						Reference
Tunis	Aerial and root	Maceration stirring	Petroleum ether dichloromethane and methanol	sequential extraction according to polarity	Not applicable		Not applicable	R. E	R. D	R. M	P. E	P. D	P. M	^27^
						C. freundii 11,041/11,042								
						E. coli 8138 and 8157								
						E. coli ATCC 25,922								
						E. aerogenes 9004								
						E. cloacae 11,050/11,051/11,053								
						K. pneumoniae 11,016/11,017								
						S. marcescens 11,056/11,057								
						P. mirabilis 11,060								
						P. stuartii 11,038								
						Salmonella sp. 11,033								
						A. baumanii 9010					1.25			
						A. baumanii 9011					0.312	0.625		
						P. aeruginosa 8131								
						P. aeruginosa ATCC 27,583,								
						S. maltophilia		< 0.78			< 0.78	< 0.78		
						Y. pseudotuberculosis 2777		< 0.78	1.25		< 78	1.25		
						E. faecalis		1.25			1.25			
						Enterococcus sp. 8153,								
						S. aureus 8146					0.625			
						S. aureus 8147					0.625			
						S. epidermidis 10,282					0.156	0.625		
						S. epidermidis 5001					0.312	1250		
						S. lugdunensis T26 A3		0.625			0.312	0.312		
						Staphylococcus warneri T12A12					0.312	1.25		
						Corynebacterium striatum T25-17					0.312	0.625		
						Mycobacterium smegmatis 5003					0.625	1.25		
						Streptococcus agalactiae T25-7		0.625			0.625			
						Streptococcus agalactiae T53C2		0.625			0.625			
						Streptococcus dysgalactiae T46C14		0.312	0.156		0.625	0.312		

Ecotype	Plant part	Extraction method	Solvent used	extract	Extract tested concentration mg/mL	Tested bacteria	Inhibition zone diameter (mm ± SD)						Minimum inhibitory concentration (mg/mL)		Reference
Egypt	shoot and root	Maceration	70% ethanol and succssive solvent fractionation according to increasing polarity Petroleum ether, chloroform, ethyl acetate, butanol, Ethanol	Crude	100 mg/mL		Shoot				Root		Shoot	Root	present study
						S. aureus ATCC29213	20 ± 0.6				16 ± 1.7		6.25 ± 0.0	3.75 ± 0.0	
						S. epidermidis ATCC 12,228	N. I				12 ± 2.8		N. I	6.25 ± 0.0	
						K. pneumoniae ATCC700603	18 ± 1.0				20 ± 0.0		12.5 ± 0.0	25 ± 0.0	
						E. coli ATCC 25,922	N.I				N.I		N.I	N.I	
						P. aeruginosa ATCC 27,853	N.I				N.I		N.I	N.I	
						S. aureus MDR	19 ± 1.0				N.I		50 ± 0.0	N. I	
						K. pneumoniae MDR	N.I				N.I		N.I	N.I	
						A. baumannii	15 ± 0.0				18 ± 1.5		12.5 ± 0.0	50 ± 0.0	
						E. cloacae	N.I				N.I		N.I	N.I	
								Petroleum ether	Chloroform	Ethyl acetate	Butanol	Ethanol	Not applicable		
				Solvent fractions		S. aureus ATCC 29,213	Shoot	18 ± 0.0	17 ± 0.0	13 ± 1.0	22 ± 3.0	8 ± 0.0			
							Root	21 ± 1.0	15 ± 0.0	14 ± 1.0	14 ± 1.0	8 ± 0.0			
						S. epidermidis ATCC 12,228	Shoot	18 ± 0.0	20 ± 0.0	13 ± 0.0	20 ± 0.0	8 ± 0.0			
							Root	20 ± 0.0	17 ± 3.0	11 ± 1.0	16 ± 1.0	8 ± 0.0			
						K. pneumoniae ATCC700603	Shoot	20 ± 0.0	8 ± 0.0	12 ± 0.0	18 ± 1.0	8 ± 0.0			
							Root	20 ± 0.0	20 ± 0.0	11 ± 1.2	14 ± 1.0	8 ± 0.0			
						E. coli ATCC 25,922	Shoot	8 ± 0.0	15 ± 0.0	16 ± 2.0	8 ± 0.0	8 ± 0.0			
							Root	13 ± 0.0	14 ± 1.0	15 ± 0.0	12 ± 2.0	8 ± 0.0			
						P. aeruginosa ATCC 27,853	Shoot	18 ± 0.0	15 ± 0.0	15 ± 2.0	15 ± 0.0	8 ± 0.0			
							Root	15 ± 0.0	15 ± 0.0	8 ± 0.0	15 ± 2.0	8 ± 0.0			
						S. aureus MDR	Shoot	18 ± 1.0	8 ± 0.0	12 ± 0.0	18 ± 1.0	8 ± 0.0			
							Root	17 ± 0.0	8 ± 0.0	11 ± 1.0	14 ± 2.0	8 ± 0.0			
						K. pneumoniae MDR	Shoot	8 ± 0.0	8 ± 0.0	11 ± 1.0	8 ± 0.0	8 ± 0.0			
							Root	14 ± 2.0	14 ± 0.0	8 ± 0.0	11 ± 1.0	8 ± 0.0			
						A. baumannii	Shoot	8 ± 0.0	8 ± 0.0	15 ± 1.0	19 ± 1.0	8 ± 0.0			
							Root	20 ± 0.0	20 ± 0.0	16 ± 1.0	24 ± 1.2	14 + 1.7			
						E. cloacae	Shoot	8 ± 0.0	8 ± 0.0	13 ± 1.0	11 ± 1.0	8 ± 0.0			
							Root	14 ± 1.0	13 ± 1.0	8 ± 0.0	8 ± 0.0	8 ± 0.0			

R. E: root petroleum ether, R.D: root dichloromethane, R. M: root methanol- P.E: aerial part root, P.D: aerial part dichloromethane, P.M: aerial part methane.

### Comparative data analysis on bacterial growth inhibition selectivity of worldwide *E. campestre* ecotypes

Published articles on *E. campestre* potency in inhibiting bacterial growth were reviewed (Table 5). This allowed uncovering genetic diversity in the plant ecotypes with respect to selectivity of bacterial growth inhibition. Variation in plant ecotypes and tested bacterial species as well as methods of extraction and plant organs noticeably affected the potency of the plant in bacterial growth inhibition. For example, the MIC of the root of the Polish ecotype was 0.9 mg/mL for *S. aureus* ATCC 29,213 while that of the Egyptian ecotype was 3.75 mg/mL i.e., 4 folds of the Polish ecotype. The ratio of MIC of aerial crude extract of the Polish to Egyptian ecotypes in inhibiting the same bacterial species was 1.9:6.25 approximately 3 folds. The extracts of the Palestinian and Algerian ecotypes have stronger antibacterial activity against Gram positive than Gram negative bacteria^24,26^ while Egyptian ecotype has potent antibacterial activity on both Gram-positive and Gram-negative bacteria.

### Plant phytochemical analysis

Phytochemical screening of the shoot and root ethanolic crude extracts of *E. campestre* indicated the presence of flavonoids, alkaloids, tannins, saponins, phenols, terpenoids, steroids, and coumarins as shown in Table 6.

## Discussion

Ecotypes of plant species are known to differ in their phytochemistry^26^. Qualitative tests for secondary metabolites in 70% ethanol extract of *E. campestre* shoot and root demonstrated the presence of flavonoids, alkaloids, tannins, saponins, phenols, terpenoids, steroids, and coumarins which may contribute to the bacterial growth inhibitory effect of *E. campestre* extracts and this agreed with previous finding of the antibacterial effects of these active compounds^28^. Flavonoids exhibit antibacterial activity through their ability to form complexes with bacterial cell walls, extracellular proteins, and soluble proteins^29^. While alkaloids exert their antibacterial effects by intercalating with bacterial DNA^30^. Moreover, the antibacterial activity of phenols is thought to be related to the site(s) and number of hydroxyl groups on their aromatic ring^31^. Furthermore, tannins display antimicrobial activity via mechanisms such as iron deprivation, hydrogen bonding, specific interactions with proteins (including enzymes and cell envelopes), and complex formation with polysaccharides^21^^,33^. As well as saponins demonstrate antibacterial properties by inducing leakage of proteins and enzymes from bacterial cells^34^. Terpenoids, the primary components of essential oils, are speculated to disrupt bacterial membranes due to their lipophilic nature^35^. Coumarins may indirectly contribute to combating infections by stimulating macrophages^36^. Finally, steroids have been reported to possess a broad spectrum of antibacterial effects^37,38^. Ten different extracts of *E. campestre* Egyptian ecotype showed bacterial growth inhibition selectivity against nine bacterial species. Petroleum ether of root and ethyl acetate of shoot inhibited the growth of nine bacterial species including *S. aureus* MDR and *K. pneumoniae* MDR. Such results indicated the significant importance of the role of natural products providing pharmaceutical solutions to the problem of developing bacterial resistance to antibiotics. The mechanism of how active compounds extracted from *E. campestre* acted on the breaking of bacterial resistance to antibiotics needs further investigation. However, differential effect and selectivity of different solvent fractions obtained from ethanolic crude extracts could be considered the first step and the key for understanding the mechanism of how natural products break bacterial antibiotic resistance. The potential presence of efflux pump inhibitors within the complex chemical composition of plant extracts has been proposed. Furthermore, the disruption of quorum sensing, a crucial mechanism for bacterial cell-to-cell communication, has also been identified as a highly promising mode of action for bioactive compounds in combating MDR pathogens^3^. Fractions generally outperformed the crude extract effect. This could be explained as at a concentration of 100 mg/mL of the root and shoot crude extracts containing different fractions varying in their amounts; this means that any of the fractions in the 100 mg is present in the crude extract by an amount less or much less than 100 mg. Testing bacterial growth inhibition of different solvent fractions at a concentration of 100 mg/mL is expected to have higher effect than when it is present as a fraction of 100 mg in the crude extract. This explains why 100 mg/mL crude extract failed to inhibit the growth of the tested nine bacterial species, while petroleum ether fraction of the shoot extract inhibited all nine bacterial species, the same for the ethyl acetate fraction of the aerial system. Similar results were obtained by Stefanović et al. (2012) and Kebede & Shibeshi. (2022), among tested extracts, ethyl acetate fraction was one of the most active in all tested microorganisms^39,40^. Genetic diversity among *E. campestre* ecotypes growing in seven countries (Table 6) possibly is behind selectivity and differential effect on bacterial growth inhibition. Active compounds concentration differs between shoot and root and also in their selective effect on bacterial species. This is clearly shown in the results of the shoot and root extracts MIC where 6.25 mg/mL of the shoot extract is capable of inhibiting the growth of *S. aureus* ATCC 29,213 while approximately half of that concentration of the root extract is enough to inhibit the growth of this bacterial species. In contrast, 50 mg/mL MIC of the crude root extract was found to inhibit the growth of *A. baumannii* while a quarter of this concentration of the shoot crude extract was found only required to inhibit the growth of the same bacterial species. Selectivity and differential effect of bacterial growth inhibition using extracts of medicinal plants are the result of interaction between plant ecotype, plant organ, extraction method and solvent used, concentration of active compounds, and tested bacterial species^3^. Statistical analysis showed significant difference between shoot and root solvent fractions in their effect on bacterial growth inhibition. This could be due to the variability in the concentration of active compounds in different plant organs. It has been reported that non-polar fractions were more active than polar ones^41^. The present work findings agree with this statement, whereas the non-polar petroleum ether extract derived from the roots showed the most significant inhibition of bacterial growth, while the polar ethanol extract from the same source exhibited the least antibacterial activity. Comparing *E. campestre* extracts of different ecotypes, those of the Palestinian and Algerian ecotypes^24,26^ showed more inhibition susceptibility of Gram positive than Gram negative bacteria. This could be due to structural differences of bacterial external membrane^42^. On the contrary, extracts of the Egyptian ecotype were found to be effective on both Gram-positive and Gram-negative bacteria. Variability in the degree of susceptibility of gram-negative and gram-positive bacteria to the plant extract is most probably a result of selective and differential effect of active compounds both in nature and concentration.

**Table 6:** Phytochemical screening of both shoot and root ethanolic crude extracts of *E. campestre.*

**Phytochemical compounds**	**Phytochemical test**	**Result**	**Photo**
Flavonoids	Alkaline reagent test	Colour change	
Alkaloids	Wagner's test	reddish-brown precipitate	
Tannins	Ferric Chloride test (FeCl_3_)	Formation of dark green colour (condensed tannins)	
Saponins	Foam test	Formation of foam or froth	
Phenols	Ferric Chloride test (FeCl_3_)	Formation of black, green colour	
Terpenoids and Steroids	Salkowski test	A reddish-brown colouration of the interface	
Coumarin	NaOH test	development of a yellow colour	

## Conclusions

The Egyptian ecotype of *E. campestre*, was found to contain active phytochemical compounds with potent inhibitory effect on bacterial growth of nine tested bacterial species including two multi drug resistant. The crude extract and its five solvent fractions varied in their inhibitory effect. Statistical analysis confirmed significant differences among bacterial species, solvent fractions, plant parts, and their interactions in bacterial growth inhibition. The present work presented Egyptian ecotype of *E. campestre* as an excellent candidate for pharmaceutical industry, particularly in the field of biocontrol of bacterial infectious diseases. However, the practical application of novel bioactive compounds remains a complex endeavor. Consequently, comprehensive in-vitro and in-vivo investigations are crucial to validate the efficacy and safety of antimicrobial compounds. Also, investigations into the precise mechanisms of action and the comprehensive pharmacokinetic and pharmacodynamic profiling of the extracts warrant significant prioritization in future research.

## Data Availability

All data generated or analysed during this study are included in this article.

## Abbreviations

ATCC: American type culture collection
ANOVA: Analysis of variance
CFU: Colony forming unit
CLSI: The clinical and laboratory standards institute
DMSO: Di-methyl-sulfoxide
E. campestre: Eryngium campestre
mg/mL: Milli gram per ml
MDR: Multiple drug resistant
MIC: Minimum inhibitory concentration
M.H.A: Muller hinton Agar
M.H.B: Muller hinton broth
N.I: No inhibition
SD: Standard deviation
t_cal_: t- calculated
t_tab_: t- tabulated
WHO: World health organization
